# Experimental and Numerical Study of AISI 4130 Steel Surface Hardening by Pulsed Nd:YAG Laser

**DOI:** 10.3390/ma12193136

**Published:** 2019-09-26

**Authors:** Giuseppe Casalino, Mahmoud Moradi, Mojtaba Karami Moghadam, Ali Khorram, Patrizia Perulli

**Affiliations:** 1 Department of Mechanics, Mathematics & Managment, Politecnico di Bari, viale Japigia 182, 70124 Bari, Italy; giuseppe.casalino@poliba.it (G.C.); patrizia.perulli@poliba.it (P.P.); 2 Department of Mechanical Engineering, Faculty of Engineering, Malayer University, P.O. Box 65719-95863, Malayer, Iran; mojtaba.kmoghadam1991@gmail.com; 3 Laser Materials Processing Research Centre, Malayer University, Malayer 65719-95863, Iran; 4 Department of Mechanical Engineering, K. N. Toosi University of Technology, P.O. Box 19395-1999, Tehran, Iran; alikhorram@ymail.com

**Keywords:** laser surface transformation hardening, Nd:YAG pulsed laser, AISI 4130 steel, FEM model

## Abstract

Laser surface transformation hardening (LSTH) of AISI 4130 was investigated by a Nd:YAG pulsed laser. Laser focal height (LFH), pulse width (LPW), scanning speed (LSS), and power (LP) varied during the experiments. The microstructure of the treated zone was characterized by optical (OM) and field emission scanning electron microscopy (FESEM). Micro-hardness was measured in the width and depth directions. Results showed that the hardness and depth of hardened layer increased by decreasing the LSS and the laser focal position (LFP), and by increasing the LPW. The results were compared with those obtained by furnace heat treatment of the same steel. Eventually, a finite element model was employed for the simulation of the LSTH of AISI 4130 steel and calculation of the heat-treated zone. The results showed that the model can predict with accuracy the temperature profile and the size and the shape of the laser hardened region.

## 1. Introduction

Surface transformation hardening processes are developed to produce wear-, corrosion-, and fatigue-resistant surfaces in order to enhance the lifespan for numerous industrial applications. There are several methods in order to improve surface properties. Among these processes, induced heat treatment and flame heat treatment are methods to improve steel surface hardness and wear resistance. While used for various industrial utilizations such as welding [1,2], drilling [3], and cutting [4], laser beam treatment is effective when a localized treatment is required [5]. In fact, laser has high accuracy and emits a centralized beam, which can be used for heat treating metal surfaces [6]. Hence, laser surface transformation hardening (LSTH) is a precise method because it offers controllable heat in the selected area in order to produce hardened surface layers. Moreover, the type of laser and the choice of material can influence the hardening process.

Interesting results were obtained in different studies on LSTH. Lo et al. [7] worked on AISI 440C LSTH. They demonstrated that this method could be better than a furnace at localized heat treatment. Dumiterscu et al. [8] investigated the fiber laser surface heat treatment of AISI D2 steel. The advantages of this process were (1) catastrophic fracture of the used carbide tools could be removed, (2) tool life was improved, (3) chatter of machining and formation of saw tooth chips could be prevented, and (4) the thrust part of the cutting force was decreased. Mahmoodi et al. carried out LSTH of AISI 420 by techniques of Nd:YAG laser; hardness in depth and width of the hardened zone, overlapping of laser treatment lines, and corrosion resistance were investigated [9]. Badkar et al. [10] analyzed the effect of LSTH on pure titanium surfaces. They concluded that the heat input had an important role in the geometrical dimension of the resulting hardened layer. In another study, Moradi et al. [11] made a high power diode LSTH of AISI 4130 steel by carbon coating and observed high micro-hardness in the carbon coated treated zone. Comparison of wear resistance of a laser processed sample and the base material demonstrated the advantage of the laser treatment.

Li et al. [12] treated AISI 1045 carbon steel by high power diode laser (HPDL) and CO_2_ laser. According to their work, performing the LSTH using HPDL with a rectangular beam shape produced a higher quality product than did the CO_2_ laser transformation hardening process with a circular beam spot. For high power diode laser surface hardened samples, the hardened depth in each sample was almost unchanged and surface melting was avoided. Otherwise, for the CO_2_ laser surface hardening samples, the results showed that melting of the surface layer occurred. Bojinovic et al. [13] simulated an LSTH process of austenitic stainless steel (50CrV4). Telasang et al. [14] studied the LSTH of AISI H13 steel, which hardened up to 800 Vickers. Additionally, wear and corrosion resistance were investigated in their study. They observed that when LSTH was conducted with a laser energy density of 75 J/mm^2^, an excellent fretting wear behavior was obtained as compared to that in as-received tool steel. Bien et al. [15] performed LSTH of C80U steel by a CO_2_ continuous wave laser. The results showed that the microstructure including highly defected fine-plate martensite with a specified number of imperfectly dissolved cementite and a small amount of retained austenite phase was obtained when C80U steel was melted by optical discharge plasma. Cordovilla et al. [16] simulated LSTH of AISI 4140 and compared the simulation results with experimental data. Guarino et al. [17] improved the fatigue life of AISI 1040 steel by a high-power diode laser treatment. Their results revealed that the laser treatment considerably raised the fatigue life of AISI 1040 steel. The mechanical properties of low carbon steel sheets subjected to laser surface hardening were investigated by Syed et al. [18]. Abboud et al. [19] studied the material responses in surface treatment of ferrous alloys by high power laser. Different laser parameters (i.e., power, laser scanning speed (LSS), spot size, and laser energy absorption) were investigated in CO_2_, Nd:YAG, and diode laser. Netprasert et al. [20] used a nanosecond pulse laser for investigation of the hardening of AISI 420. The results showed that the micro-hardness increased from 242 HV to 1700 HV and the depth of the hardened layer was in the 60–80 µm range. Yazici et al. [21] investigated the effect of different processing temperatures on wear properties of R260 grade rail steel. They studied the mechanical properties, surface hardness, tribological properties, and microstructure of high-power diode laser treated specimens. Lesyk et al. [22] compared the effects of the ultrasonic impact treatment with those of laser heat treatment on the AISI D2 steel. Moreover, the twi treatments were combined and it was found that the surface wear losses were decreased by 77%. Barka et al. [23] used a finite difference method (FDM) for simulating the LSTH process of AISI 4340 steel. They compared the results of the simulation with experimental data. Casalino [24] used the finite element model (FEM) analysis to study the laser treatment for recovery of deformation that was induced by surface laser hardening. Recently, Athanasiou revealed brittle-fracture statistics from intermittent patterns formed during femtosecond laser exposure [25]. Moreover, he designed a monolithic micro-tensile tester for investigating the silicon dioxide polymorph micromechanics, which was fabricated and operated using a femtosecond laser [26].

The laser surface transformation hardening process of steel is an encouraging industrial application considering the wide employment of steel in the industries. In fact, low alloy carbon steel (AISI 4130) is a heat treatable steel, which is used in several industries (i.e., petroleum, gas, petrochemicals, food, and pharmaceuticals) [27]. Especially, Nd:YAG laser surface transformation hardening of AISI 4130 is used for areas that require a low width of hardness and high precision. During the LSTH process, the phases transformation of ferrite and austenite to martensitic improves the surface hardness. In particular, because of the cooling, only the austenite phase transforms into martensite.

In this paper, LSTH of AISI 4130 was performed by Nd:YAG pulsed laser with a maximum power of 400 W. The effects of laser focal point position, laser pulse width, and LSS were investigated after LSTH of AISI 4130 alloy steel. The microstructure was analyzed by optical microscopy (OM) and scanning electron microscope. Moreover, Vickers micro-hardness tests were carried out in the transversal section. The results of LSTH were compared with those obtained by furnace heat treatment.

Eventually, a finite element model (FEM) was employed for the simulation of the LSTH of AISI 4130 steel. For this purpose, a cylindrical heat source was modelled to simulate the surface hardened. A continuous cooling transformation (CCT) curve for AISI 4130 steel was employed for the validation of the numerical model. The results show that the model can predict with accuracy the temperature profile and the size and the shape of the laser hardened region.

## 2. Experimental Work

Samples of 65 mm diameter and 10 mm thickness were machined and treated for laser surface and furnace hardening. Table 1 shows the AISI 4130 chemical composition (the median three of XRF measurements). XRF (X-ray fluorescence) is a non-destructive analytical technique used to determine the elemental composition of materials.

The experiments were performed by a 400 W average laser power pulsed Nd:YAG laser (model IQL-10, Paya Parto, Tehran, Iran). The available range for the pulse frequency, the pulse duration, and the pulse energy were 0.2–20 ms, 1–1000 Hz, and 0–40 J, respectively. The average power of the laser did not exceed 700 W. Three lenses (75 mm focal length) were used in the focusing optical system, and the minimum spot size was 250 mm. Average power in each experiment and pulse energy were measured by LA300W-LP joule meter (Edu-lab, Karoo Close, Bexwell Business Park, Norfolk, England) and 5000W-LP Ophir (Ophir Photonics Group-Ophir-Spiricon, North Logan, UT, USA). The plan of the experiment for LSTH is shown in Table 2.

**Figure 1:**
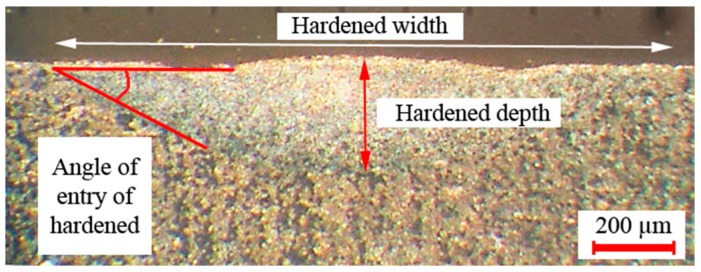
Width of hardened case, depth of hardened case, and angle of entry angle of hardened areas.

The pulse frequency was kept at 15 Hz. After LSTH, the samples from the medium of the hardening line were sectioned by wire cut EDM (electrical discharge machining). The samples were prepared with standard metallographic grinding and polishing techniques and etching to observe the microstructures with the following reagent: 2% Nital’s solution (ethanol 98%, nitric acid 2%) according to ASTM E 407. Leica MEF 4A optical microscopy (Leica Microsystems, 1700 Leider Lane, Buffalo Grove, IL, USA) with 50–100X magnification and the ImageJ software (LOCI, University of Wisconsin, Madison, WI, USA) was used for measuring the geometry features of the hardened case, i.e., width and depth of hardened case, angle of entry of hardened area; see Figure 1.

**Figure 2:**
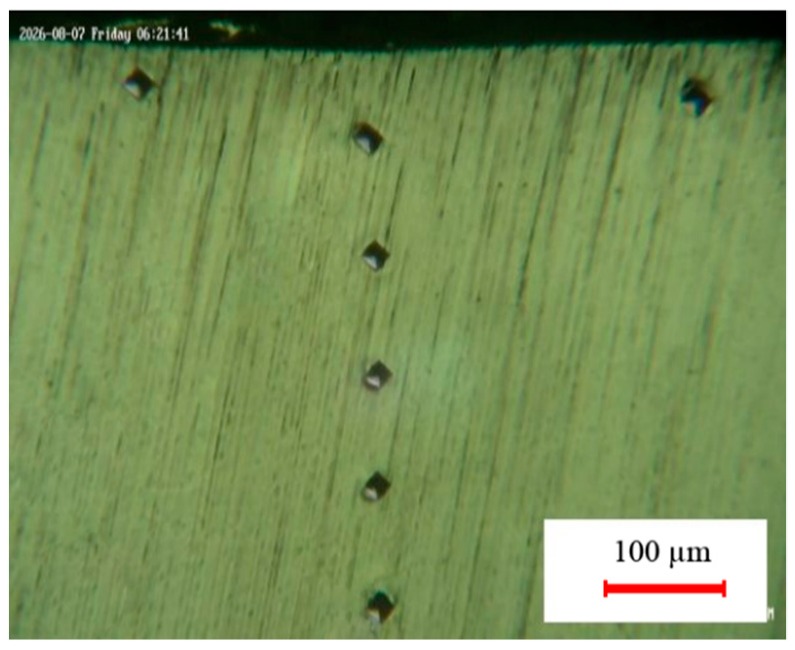
Micro-hardness indenters (Vickers) in width and depth.

**Figure 3:**
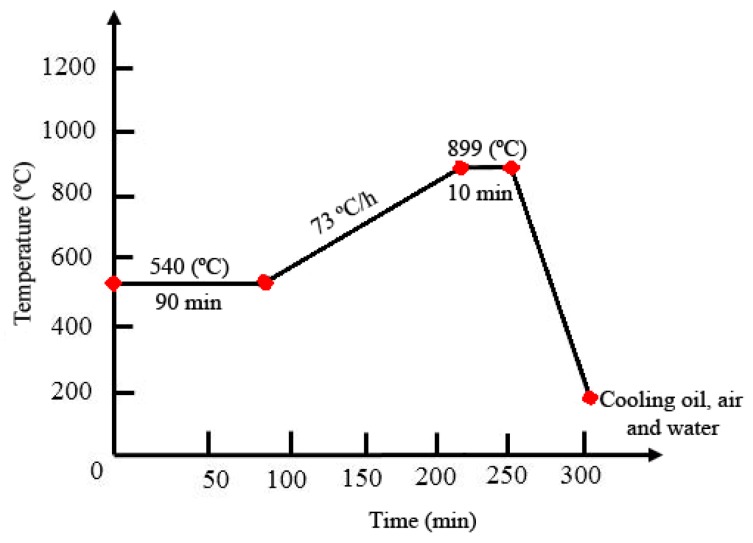
The cycle of AISI 4130 of furnace hardening heat treatment [27].

OM and field emission scanning electron microscopy (FESEM) were applied for investigating the hardened layer microstructure. The percentage of the ferrite phase in the average of the structure of the hardened zone was measured by Celemex software (manufacturer, city, state, country). A micro hardness V-test-analog tester was used for 30 s dwell time with 100 gr maximum load. Vickers tests were repeated three times for each treatment condition. The micro-hardness profiles through the line from the center of the hardened zone to the untreated zone in depth and width directions of the hardened area were plotted for each sample. Figure 2 shows the cross-sectional view of micro-hardness indenters in the surface and depth of the hardened cases. Furnace heat treatment according to the cycle shown in Figure 3 was performed [28,29]. The AISI 4130 was preheated to 540 °C for 90 min and heated to 899 °C at a rate of 73 °C/hour and was retained for 10 min. The hardened cases were quenched in water, air, and oil.

## 3. Numerical Model

Nowadays, the finite element method (FEM) has been developed to reduce solution time and to predict thermal distortions, residual stress, and metallurgical change during the heat treatment. Therefore, in FEM simulation, in order to obtain a satisfactory process representation, the choice of the heat source model is very important. Moreover, validation and calibration of the model are essential to define heat source parameters correctly in order to establish a link between numerical and experiments results. Many studies have been conducted with different heat source models [30]. Rosenthal [31] suggested a mathematical model of power density distribution. He proposed both punctual and line heat source. Pavelec et al. [32] developed a Gaussian power density distribution on the surface of the workpiece. Other researchers [33] proposed a conical heat source model.

In this paper, the finite element model was built using the Simufact Welding 8.0 software (MSC Software, Hamburg, Germany). Thermo–physical and mechanical properties have important influences on the results of FEM simulation. Thermal conductivity was taken as 42.7 W/mK, while liquidus temperature and solidus temperature were taken 1733 K and 1693 K, respectively. These properties are displayed in Table 3.

**Figure 4:**
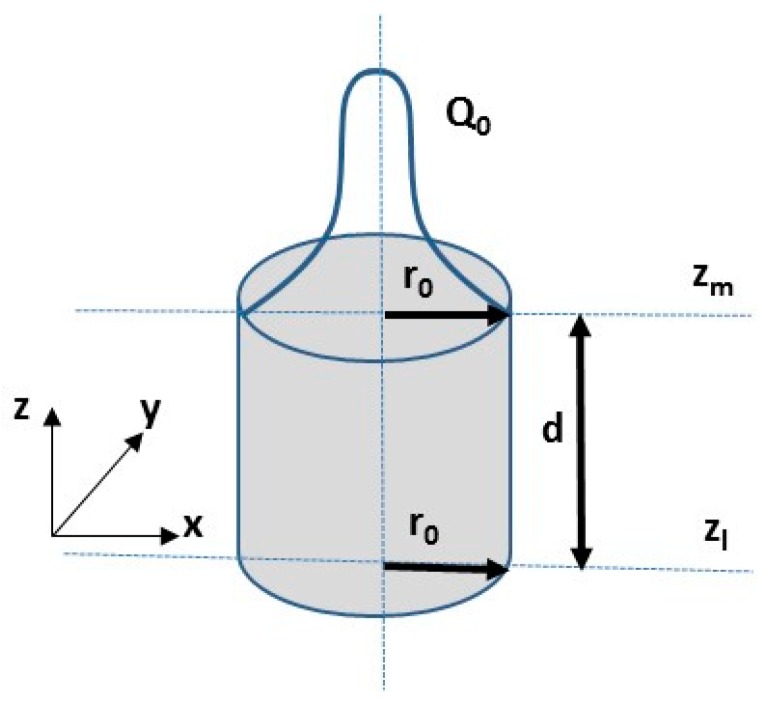
Cylindrical heat source model.

In order to describe the laser beam power distribution, the laser beam was modelled as a cylindrical moving heat source with Gaussian heat flux distribution simulation, as showed in Figure 4 [34,35,36].

The power density distribution was as follows:

(1) $$Q_{r}=Q_{o}\exp\left(\frac{-r^{2}}{r_{o}^{2}}\right)$$

where Q_r_ defines heat source intensity, Q_0_ represents the maximum intensity, while r_o_ defines the upper and the lower radius in the upper plane at z = z_u_ and in the lower plane at z = z_l_, respectively.

The radius r is given by

(2) $$r=\sqrt{x^{2}+y^{2}}.$$

## 4. Results and Discussion

In this study, the effects of Nd:YAG laser parameters (i.e., LSS, laser power, and stand-off distance) on AISI 4130 steel in the surface hardening process was studied. To investigate the surface properties, geometrical dimensions of the hardened zone, microhardness distribution in width and depth of LSTH, percentage of the ferrite phase, and microstructure of the hardened surface were investigated.

### 4.1. Hardened Case Geometry

**Figure 5:**
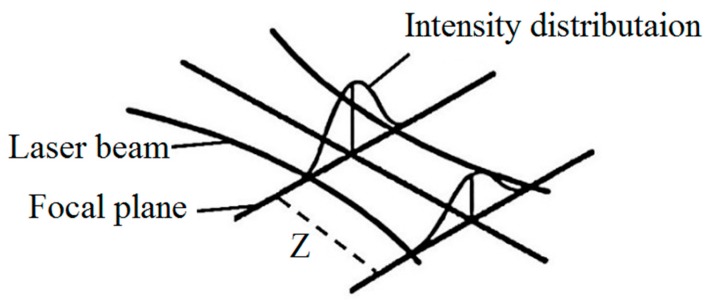
Schematic of laser beam Gaussian distribution [35].

The shape of the hardened zone was affected by the shape of the Gaussian laser energy distribution, as shown in Figure 5. The Nd:YAG laser used in this study had a Gaussian mode in which the maximum energy was focused at the middle of the laser beam. The laser energy reduced aside the middle of the laser beam. By increasing the distance from the focal plane (see z in Figure 5), not only the energy was reduced, but also the laser beam diverged.

**Figure 6:**
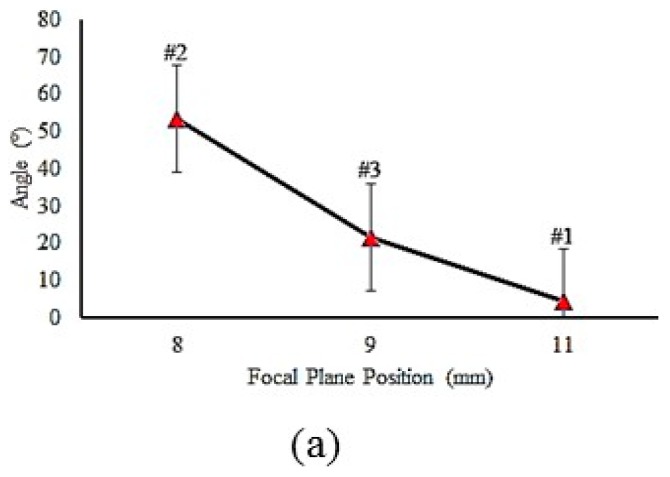

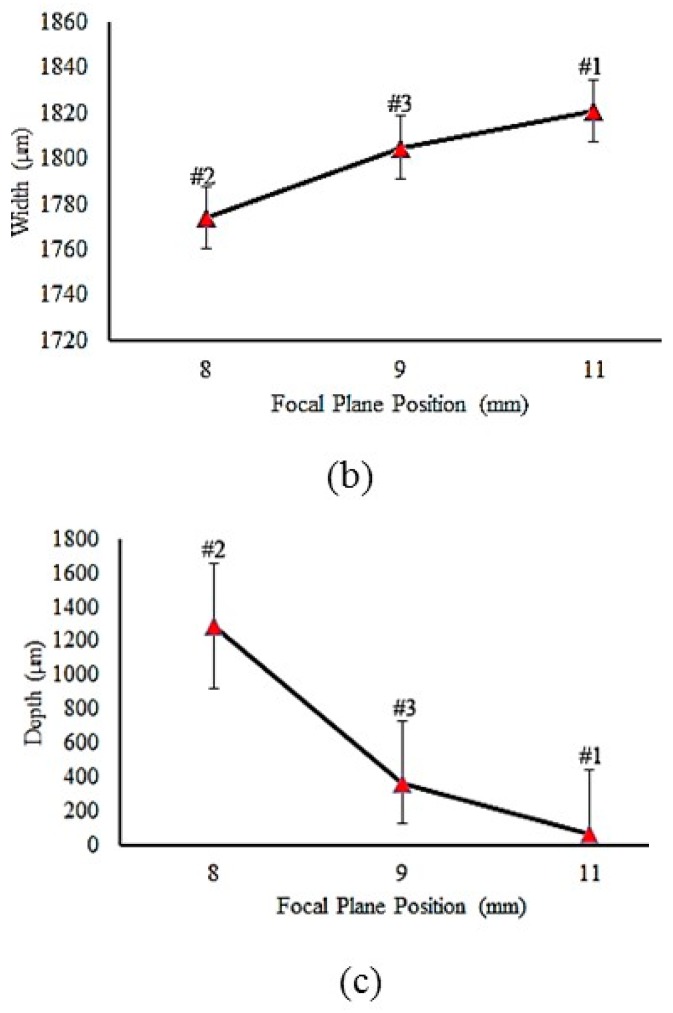
Influence of the focal plane position on LH (Laser Hardening Process) profile geometry. (**a**) Focal plane position on angle (samples #1, #2 and #3) (**b**) Focal plane position on width (samples #1, #2 and #3) (**c**) Focal plane position on depth (samples #1, #2 and #3).

**Figure 7:**
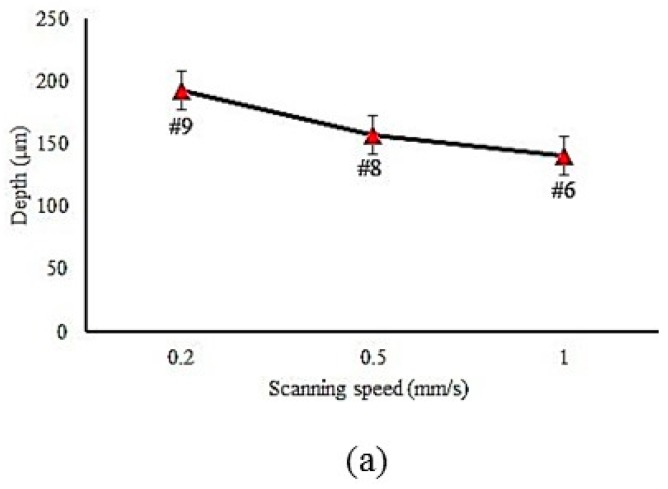

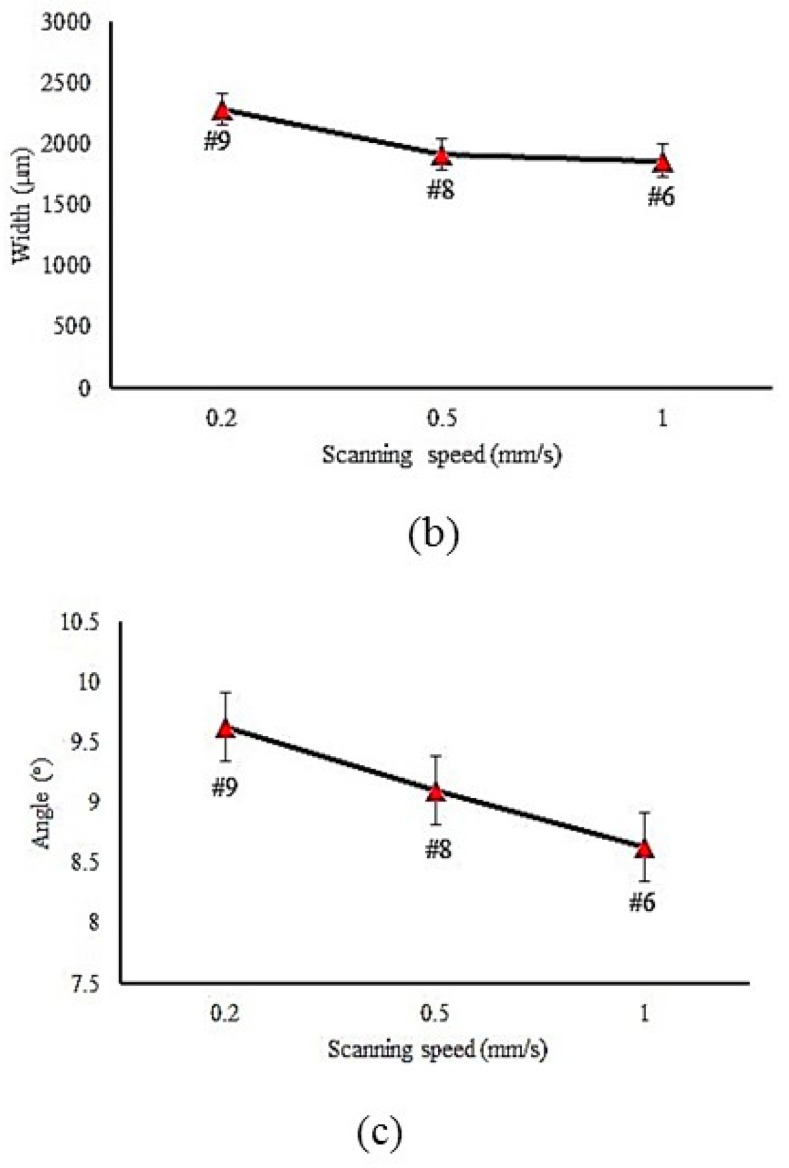
Influence of the laser scanning speed (LSS) on LH profile geometry. (**a**) LSS on depth (samples #6, #8 and #19); (**b**) LSS on width (samples #6, #8 and #9); (**c**) LSS on angle (samples #6, #8 and #9).

**Figure 8:**
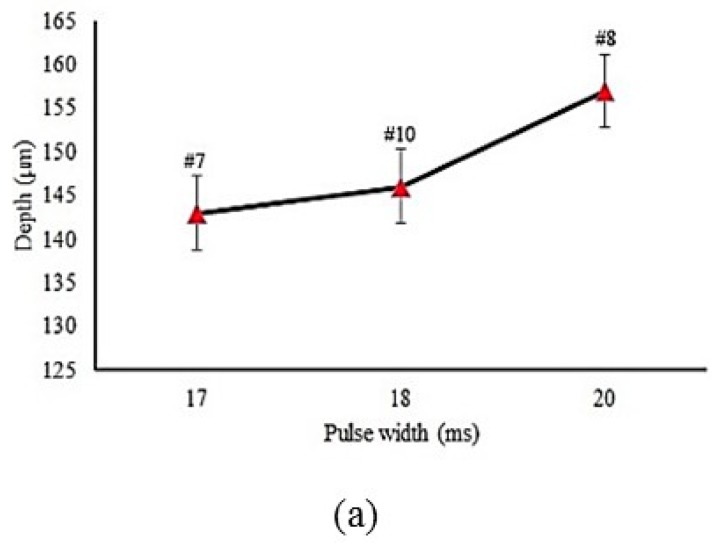

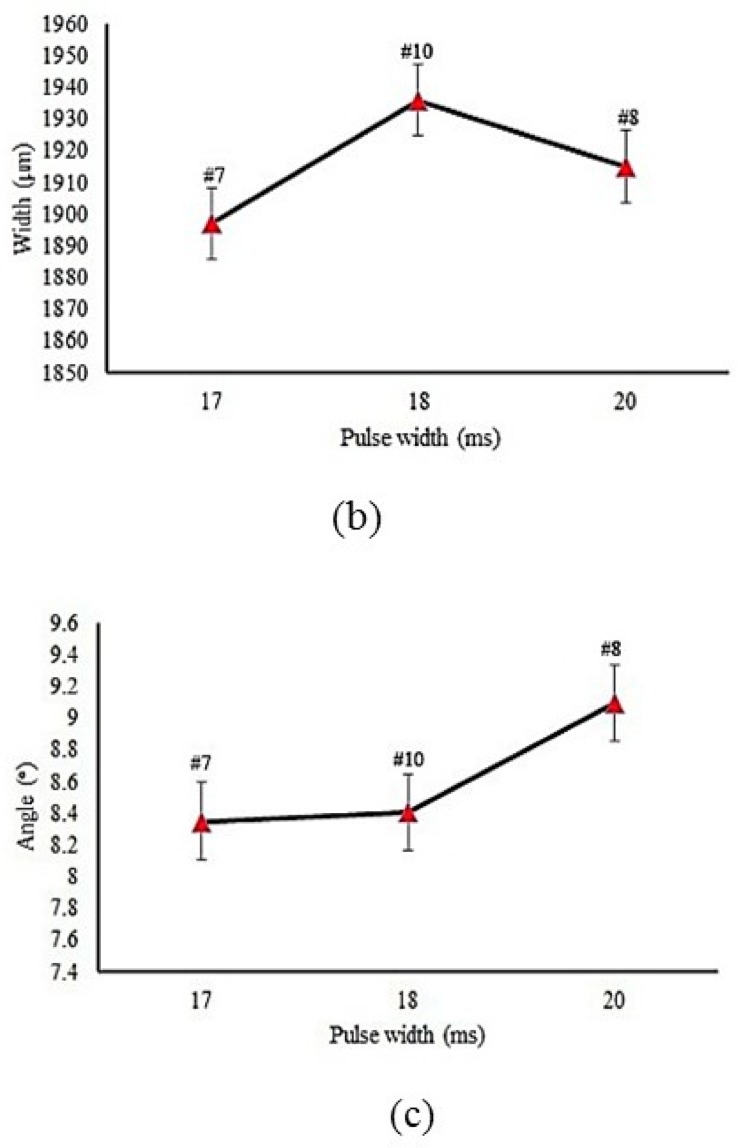
Influence of the pulse width on LH profile geometry. (**a**) Pulse width on depth (samples #7, #8, and #10); (**b**) pulse width on width (samples #7, #8, and #10); (**c**) pulse width on angle (samples #7, #8, and #10).

The input parameter effects on the hardened zone geometry are plotted in Figure 6, Figure 7 and Figure 8. The focal plane position had a direct effect on the width and a reverse effect on the depth and angle [37]. This phenomenon can be explained by analysis of the laser beam distribution displayed in Figure 5. In fact, by increasing the focal plane position (z distance in Figure 5), the laser beam transferred lower energy to the piece [38,39,40], which caused a wider and shallower hardened layer. While the width of the hardened layer increased and the depth decreased, the angle became smaller. Figure 7a–c account for the influence of LSS on the geometrical dimensions of the hardened layer. It consisted in the fact that the lower LSS caused wider and deeper case hardening with a bigger layer angle. That can be explained with LSS reduction, which increased the interaction time between the material and the laser beam. Therefore, more heat energy was transferred to the surface of the samples and wider and deeper hardened layers were achieved. Sample #9 had a deeper hardened layer, which was 193 µm, and 2284 µm width. Although samples #2 and #3 had the maximum depth and hardness, i.e., the surface of these samples was melted, they were not the best ones, while transformation hardening was desired. The melting surface was due to lower focal plane position distance (8 mm and 9 mm), which led to higher energy concentration and then the energy caused the melt of the material surface.

The pulse energy is calculated by Equations (3) and (4):

(3) $$E=\frac{P_{\mathrm{Average}}}{F_{\mathrm{Pulse}}}$$

(4) $$P_{\mathrm{Peak}}=\frac{E}{W_{\mathrm{Pulse}}}$$

where E is the pulse energy (joule), *P*_Average_ is the average power (Watts), F_Pulse_ is pulse frequency (Hz), P_Peak_ is the peak power (kW), and *W*_Pulse_ is the pulse width (ms).

The influence of pulse width on the depth, width, and hardened layer angle is shown in Figure 8a–c, respectively. Increasing the pulse width led to increases in the depth and hardened layer angle. When the pulse width increased, the laser energy increased. Therefore, more heat was transmitted to the sample surface. This phenomenon caused a larger hardened area (see Equations (2) and (3)).

By comparison, in samples #5 and #6, in which only the value of the pulse width differed, it could be observed that the higher the pulse width, the higher width and depth in the geometrical dimension and harder layer was obtained. More heat to the surface of the sample caused higher hardness and a larger hardened area. This trend was the same for sample #7 and sample #8.

### 4.2. Micro-Hardness Distribution

**Figure 9:**
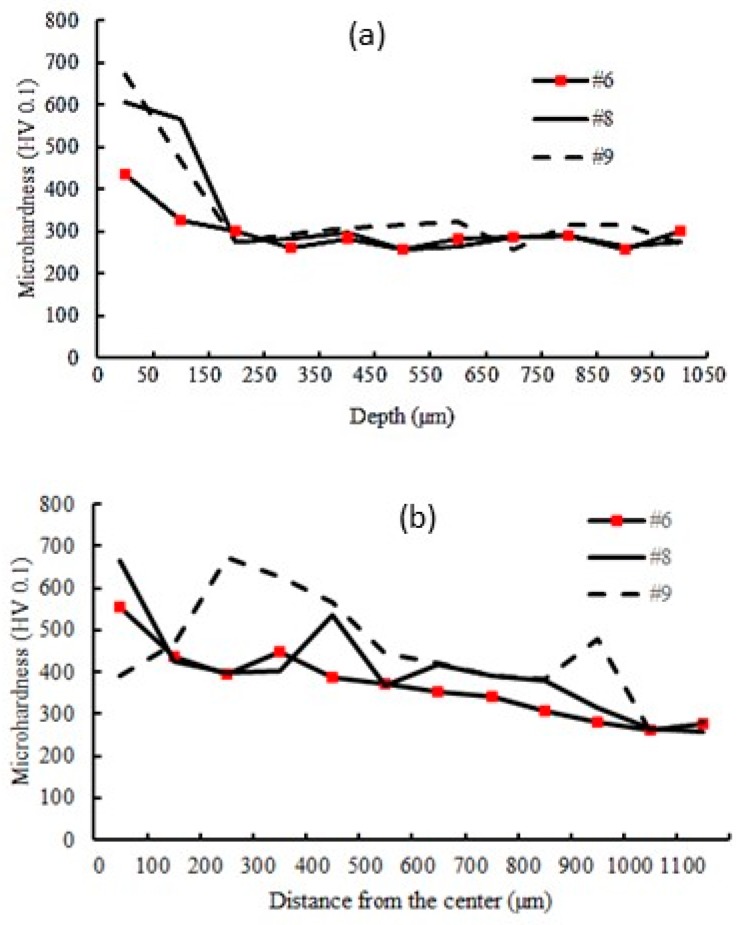
Micro-hardness profile in (**a**) depth and (**b**) width of the hardened laser layer (samples #6, #8, and #9 different LSS).

Figure 9a,b displays the depth and width of microhardness profile, respectively.

LSS is one of the most significant parameters in many laser materials processes [41] and it also applies to LSTH. Samples #6, #8, and #9 had the same input parameters with different LSS. In Section 4.1, Figure 7a,b deeply explained the effect of varying the LSS in the LSTH process. By the same trend, the effect of LSS on micro-hardness distribution can be observed in Figure 9.

The lower LSS left more time to the laser beam to heat the material surface. It is clearly shown in Figure 9 that a higher level of hardness was produced with the lower LSS, sample #9. In Figure 6c and 7a the maximum value of the width and depth of the hardened zone occurred in the lowest LSS. In this study, the maximum surface hardness without material melting was 673 HV.

**Figure 10:**
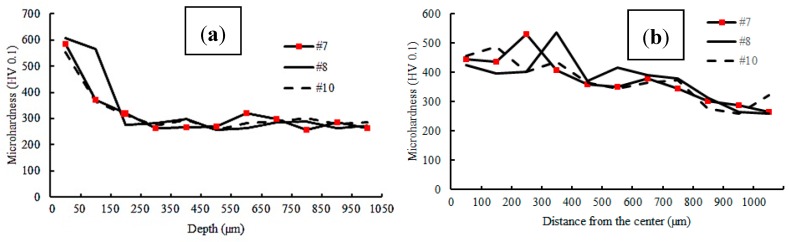
Micro-hardness profile in (**a**) depth and (**b**) width of the hardened laser layer (samples #7, #8, and #10); see Figure 2.

The pulse width plays also a major role in material laser processing [42,43,44,45]. The effect of pulse width on micro-hardness distribution is shown in Figure 10. When the pulse width increased, the micro-hardness value in the hardened layer increased as well. The micro-hardness value in sample #8 reached 665 Vickers. A high amount of energy was transferred to the surface of the sample, which caused higher hardness.

The micro hardness deviation from base metal (MHD) is derived from Equation 5:

(5) $$\mathrm{MHD}=\sum_{i=1}^{n}\frac{(x_{i}-x_{b.m}){}^{2}}{n}$$

where x_i_ is the depth or width areas that Micro-hardness was measured, x _(b.m)_ is row material micro-hardness, and n is known as the number of areas that their hardness was measured. In this paper n is, 10 and x _(b.m)_ is equivalent to 256 Vickers (hardness of AISI 4130). In laser hardening process the higher MHD is desired while a higher MHD value represents a more uniform hardness in the hardened area. By comparing MHD values presented in Table 2, which were calculated by Equation (5), sample #9 had the higher values among the transferred hardening samples. The higher MHD showed better and more uniform distribution of the hardened profile.

### 4.3. Microstructure Analysis

**Figure 11:**
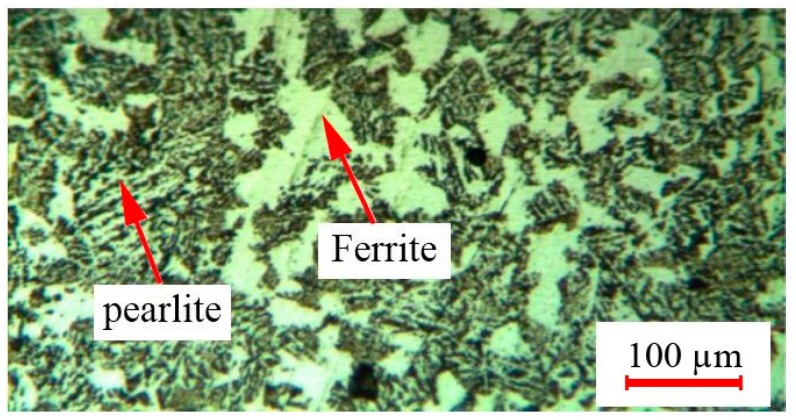
AISI 4130 base metal structure.

To investigate the influence of the LSTH process on the microstructure, the metallographic analysis was conducted by OM and FESEM. Figure 11 shows the microstructure of the low alloy carbon of AISI 4130 base material, which contained ferrite and pearlite distributed in the martensitic phase.

**Figure 12:**
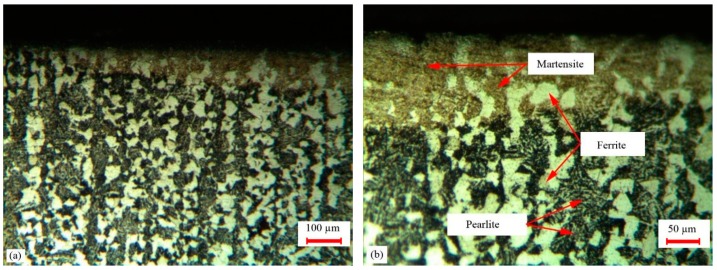

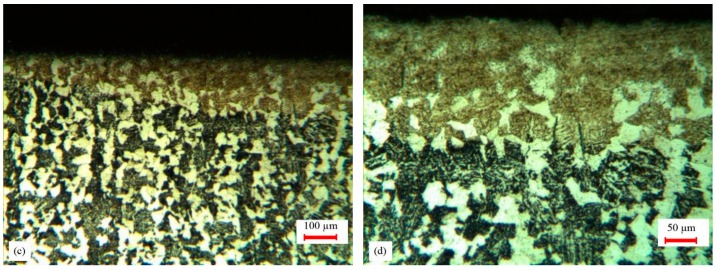
Microstructure of the LH zone of samples #5 and #9. (**a**) 50× of sample #5; (**b**) 100× of sample #5, (**c**) 50× of sample #9; (**d**) 100× of sample #9.

Figure 12 illustrates the microstructure of the best LH sample (#9). In sample #9 the value of the ferrite phase was less than other samples. The ferrite phase caused a reduction of the hardness. Thus sample #9 with the lower ferrite percentage had higher hardness. The high pulse width and low LSS led to more heat energy and caused more martensite phase and more surface hardness. Due to the uncontrollable cooling rate, the presence of the retained austenite was possible. As seen in Figure 12, the ferrite phases were bright in the martensite phase, which formed by austenite during air cooling. In the hardened zone the martensite had fine grain (see Figure 12). High cooling rates and lack of enough time for a solution led to incomplete ferrite, which remained in the hardened structure. This phenomenon caused low hardness [20]. In the LH zone, some ferrite phase with some retained austenite could be observed (see Figure 12).

**Figure 13:**
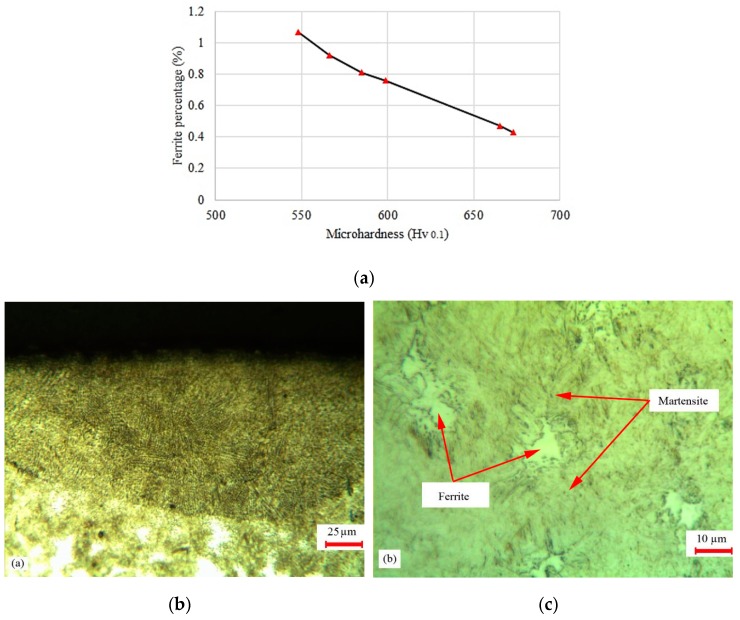
Mixture of ferrite and martensite phase and the percentage profile in maximum surface hardness. (**a**) Percentage profile in maximum surface hardness; (**b**) 200× of sample #9; (**c**) 500× of sample #9.

Figure 13a displays the influence of ferrite percentages on the maximum microhardness of different LH samples. As seen, decreasing the ferrite percentage resulted in increasing the maximum surface hardness. In sample #9, the percentage of the ferrite phase was 0.43% in the center of the hardened zone, which was less than the value of the other samples. In Figure 13b,c the mixture of the ferrite phase and the martensite phase is also displayed.

The comparison of traditional hardening and the LSTH process are shown in Table 4. After furnace hardening, samples were quenched in 3 different environments (water, air, and oil). In water, micro-cracks were created and hardness in the air was very low (about 421 Vickers). After oil quenching, the sample showed hardness as high as 572 Vickers). Since Nd:YAG LSTH treatment produced 673 Vickers, the increment in hardness was 18% higher with respect to the furnace treatment.

**Figure 14:**
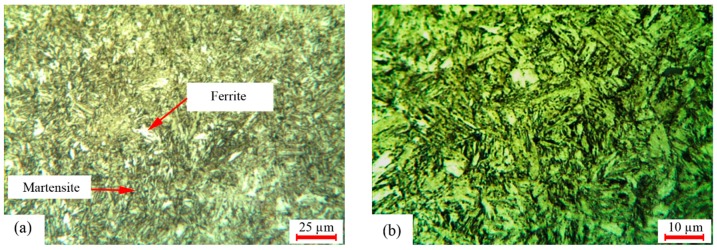
(**a**) AISI 4130 microstructure of furnace hardened quenched in oil magnitude of 200×; (**b**) magnitude of 500×.

Figure 14 presents the microstructure image of the furnace heat treated sample cooled in oil. According to metallographic evaluation, the martensite phase in the furnace hardening quenched in the oil sample (Figure 14) was uniform and the base phases of AISI 4130 was ferrite–perlite. In addition, there was an unsolved ferrite phase.

**Figure 15:**
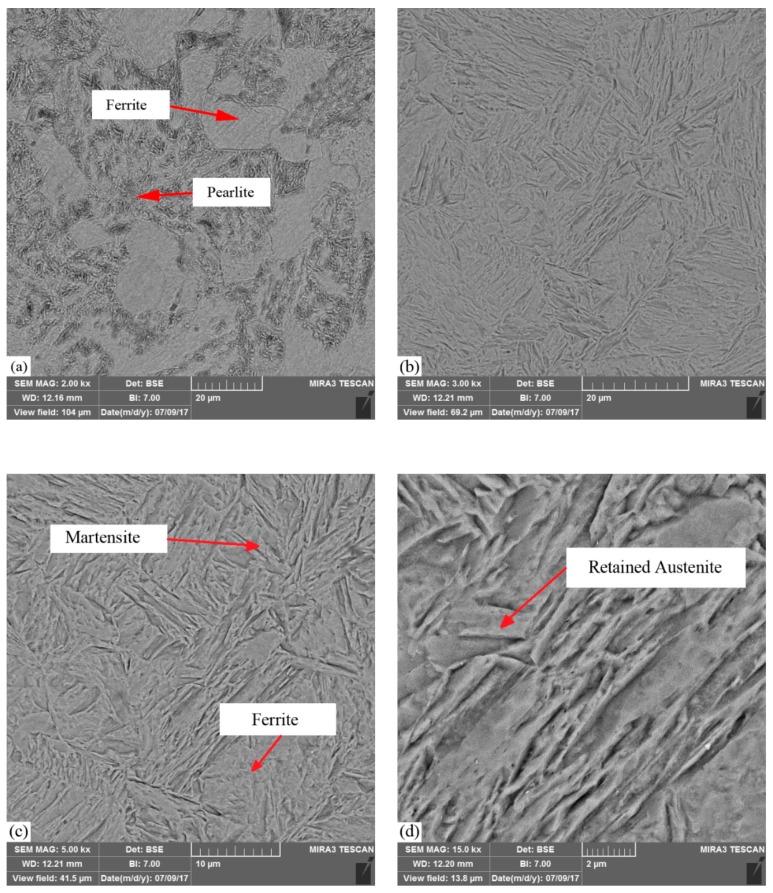
Field emission scanning electron microscopy (FESEM) images of the LH zone and base material of AISI 4130. (**a**) Base material 2 KX; (**b**) hardened zone 3 KX; (**c**) hardened zone 5 kx; (**d**) hardened zone 15 KX.

The microstructure of the laser surface hardening (sample #9) is illustrated in Figure 15 in different magnitude by using FESEM. These pictures were taken at the center of the hardened zone, which was 50 micrometers under the sample surface. Figure 15b shows the ferrite and martensite phases in the hardened zone. In Figure 15d, retained austenite in the formed martensite phase is depicted.

The brighter zone was ferrite, which was uniform, and the darker region was the perlite phase in the base material of AISI 4130. In Figure 15b–d the needle-shaped plate martensitic phase (dark needles), some retained austenite (bright zones), and some ferrite phase can be observed.

## 5. Calibration and Validation of the Numerical Model

**Figure 16:**
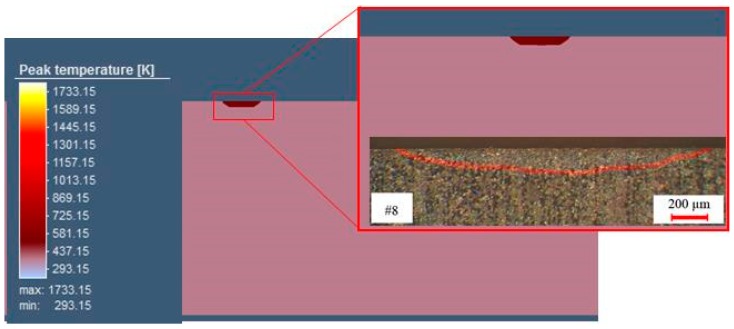
Cross section of sample #8 and calibration of the model.

The calibration of the FEM model was achieved by comparing the micrographic cross section after the heat treatment. Figure 16 displays the comparison between the simulated and experimental hardened zone for sample #8. The geometry of the hardened zone was like that achieved in the laser surface transformation hardening.

The dimension of the hardened zone can be seen in Table 5. The numerical model for the sample #8 was in good agreement between the predicted and the measured study. The results showed that a cylindrical heat source used in the simulation was capable of modeling with precision the size and the shape of the hardened zone.

In order to obtain a good hardened zone profile in the FEM analysis, the heat source parameters had to be manipulated to calibrate the hardened region shape. This phase was very important in order to obtain the accurateness of the simulation. The cylindrical heat source model was identified by three parameters (see Figure 4). They were the heat source upper and lower radius (r_o_), and the cylindrical heat source depth (d). Table 6 displays the adopted heat source parameters at the end of the calibration process.

**Figure 17:**
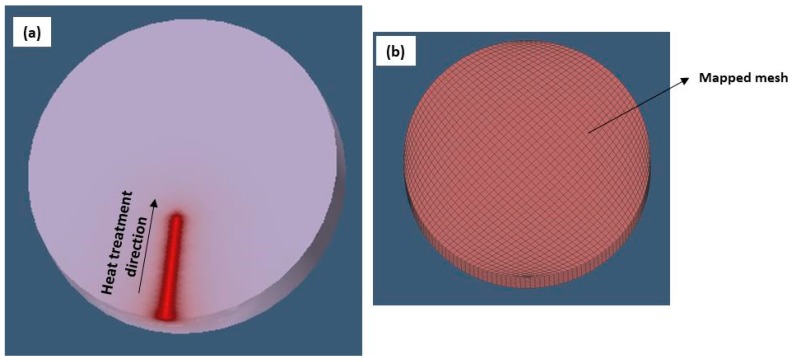
The temperature distribution in the heat treatment direction. (**a**) The temperature distribution in the hardening direction (**b**) The mesh used in the FEM simulation

The temperature distribution in the hardening direction is shown in Figure 17a. During the simulation, the temperature reached about 1400 K by ensuring the phase transformation in the hardened region. Mesh size was essential to achieve the precision of the temperature distribution in the regions with high thermal gradients. The mesh used in the FEM simulation is shown in Figure 17b. The mapped mesh in the geometry had 12,800 volumetric elements and 1024 nodes. The mesh was applied uniformly in the sample volume.

**Figure 18:**
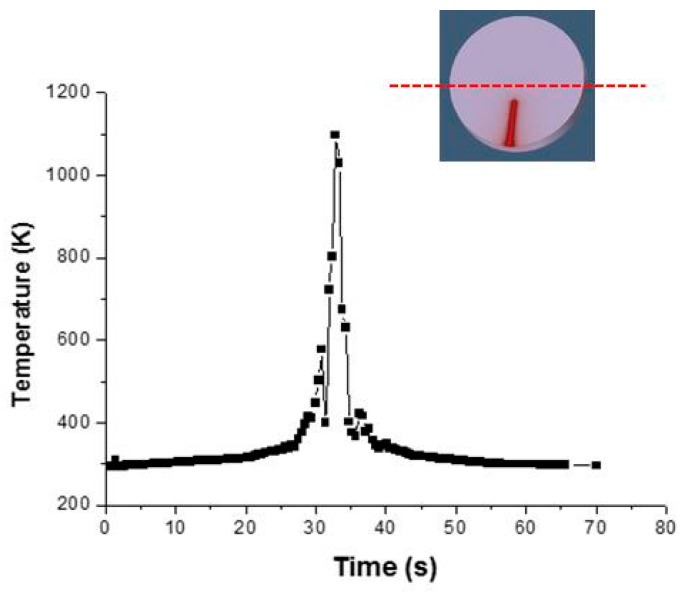
Thermal cycle measured in different points along a diameter perpendicular to the direction of the heat treatment.

**Figure 19:**
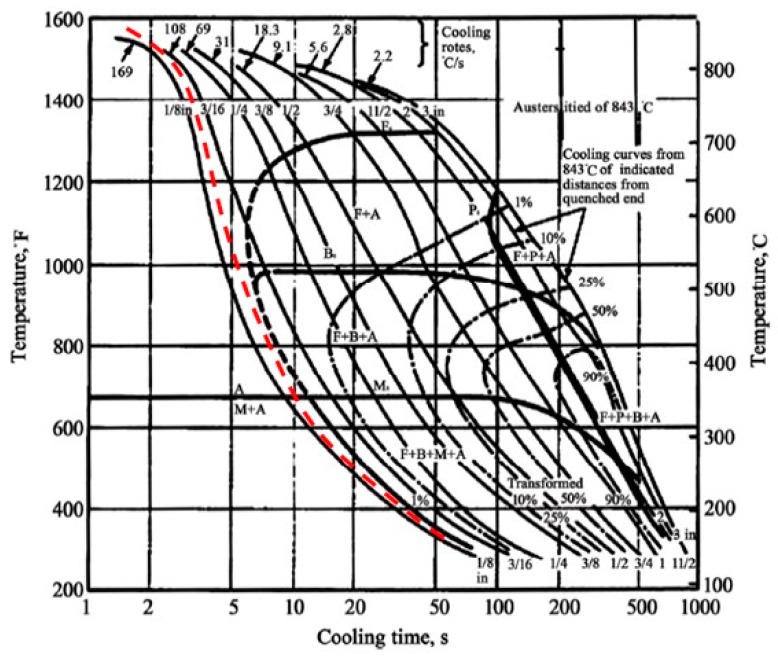
Continuous cooling transformation (CCT) diagram for AISI 4130 and cooling speed during the heat treatment (dotted curve).

The model was validated comparing the thermal cycle (Figure 18) with CCT curves to predict the microstructures in the hardened zones of AISI 4130 steel. Based on the CCT diagram, at high cooling rates (v = 130 °C/s) a martensitic microstructure with retained austenite was observed. The CCT curve is shown in Figure 19 [46].

## 6. Conclusions

The present work has reported the effect of process parameters of laser surface hardening of AISI 4130 steel. The metallurgical analyses showed that the hardened zone was a mixture of martensitic and ferritic phases with retained austenite. Moreover, a finite element model was utilized to simulate the temperature distribution in the heat treatment direction and to calculate the heat-treated zone. The FEM simulation was satisfactory.

The following considerations were pointed out:

(1) Pulse width has a significant effect on the pulsed laser surface hardening process. With increasing pulse width, the hardness value and hardened depth increase.
(2) Decreasing LSS causes the hardness value to increase. There is a lower ferrite phase in the laser hardened layer.
(3) By decreasing the focal plane position, laser energy density increases, which causes the hardness value to increase. The surface of samples #2, #3, and #4 were melted due to lower focal plane position distance, which led to higher energy concentration.
(4) Sample #9 with the higher martensitic phase percentage had more hardness and higher MHD values. Additionally, in the laser hardened zone, some ferrite phase with some retained austenite was observed.
(5) The maximum hardness value for the LH cases is 673 Vickers while this value for the furnace hardened cases is 572 Vickers, because there is a minor ferrite phase in LH cases compared to furnace hardened cases.
(6) The results of the numerical model show that the heat source model can predict with accuracy the temperature profile and the size and the shape of the hardened region. The continuous cooling transformation (CCT) curve for AISI 4130 steel confirmed the validation of the numerical model at metallurgical transformation.

## Figures and Tables

**Table 1:** Chemical composition of AISI 4130 steel (Wt.%).

Element Name	Cr	Fe	S	Al	Cu	Ni	Mn	V	S	C	Mo	Si
Weight Percent	0.03	0.024	0.06	0.05	0.87	0.012	0.03	0.25	0.25	0.3	1.01	Balance

**Table 2:** Experimental setting and results of laser surface hardening by Nd:YAG laser.

No.	Input Variables				Output Responses							Surface Melting
	Focal Plane Position (mm)	Pulse Width (ms)	Laser Power (W)	Scanning Speed (mm/s)	Depth of Hardness (µm)	Width of Hardness (µm)	Angle of Entry (°)	Maximum Hardness (hv)	Ferrite Percent (%)	MHD in Depth	MHD in Width	
1	11	15	130	1	71	1821	4.37	397	14.05	1592	21,474	-
2	8	15	130	1	1291	1774	53.3	698	0.25	19501	19,768	yes
3	9	15	130	1	360	1805	21.67	690	0.32	17,040	21,115	yes
4	9	17	135	1	271	1768	17.07	681	0.4	9242	7848	yes
5	11	17	135	1	100	1837	6.14	548	1.07	1932	22,042	-
6	11	20	155	1	141	1862	8.63	566	0.92	4730	26,435	-
7	11	17	135	0.5	143	1897	8.35	585	0.81	5520	34,454	-
8	11	20	155	0.5	157	1915	9.1	665	0.47	7300	37,136	-
9	11	20	155	0.2	193	2284	9.63	673	0.43	8563	43,202	-
10	11	18	140	0.5	146	1936	8.41	599	0.76	6430	39,079	-

**Table 3:** Mechanical and thermo–physical properties of AISI 4130.

Property	AISI 4130
Young modulus (GPa)	190
Density (g/cm^3^)	7.85
Thermal conductivity (W/mK)	42.7
Liquidus temperature (K)	1733
Solidus temperature (K)	1693

**Table 4:** Laser surface transformation hardening (LSTH) and furnace micro-hardness.

Heat Treatment Cycle	Furnace Hardening Heat Treatment	LSTH
Cooling in oil	572 Vickers	-
Cooling in water	681 Vickers	-
Cooling in air	421 Vickers	-
LSTH (#9 Table 2)	-	673 Vickers

**Table 5:** Comparison of experimental and numerical dimensions (mm) of hardened zone.

Sample #8	Experimental Data (mm)	Numerical Data (mm)
Width of hardness (mm)	1.9	1.8
Depth of hardness (mm)	0.1	0.2

**Table 6:** Cylindrical heat source parameter.

Meaning	Symbol	Value
Cylindrical heat source upper–lower radius (mm)	r_o_	0.1
Cylindrical heat source depth (m)	d	0.4
